# Discovery of Specific Inhibitors for Intestinal *E. coli* *β*-Glucuronidase through *In Silico* Virtual Screening

**DOI:** 10.1155/2015/740815

**Published:** 2015-03-09

**Authors:** Ta-Chun Cheng, Kuo-Hsiang Chuang, Steve R. Roffler, Kai-Wen Cheng, Yu-Lin Leu, Chih-Hung Chuang, Chien-Chaio Huang, Chien-Han Kao, Yuan-Chin Hsieh, Long-Sen Chang, Tian-Lu Cheng, Chien-Shu Chen

**Affiliations:** ^1^Graduate Institute of Pharmacognosy, Taipei Medical University, 252 Wu Hsing Street, Taipei 11031, Taiwan; ^2^Institute of Biomedical Sciences, Academia Sinica, 128 Section 2, Academia Road, Nankang, Taipei 11529, Taiwan; ^3^Institute of Biomedical Science, National Sun Yat-Sen University, 70 Lienhai Road, Kaohsiung 80424, Taiwan; ^4^Department of Pharmacy, Chia Nan University of Pharmacy and Science, 60 Section 1, Erh-Ren Road, Tainan 71710, Taiwan; ^5^Department of Biomedical and Environmental Biology, Kaohsiung Medical University, 100 Shih-Chuan 1st Road, Kaohsiung 80708, Taiwan; ^6^Graduate Institute of Medicine, Kaohsiung Medical University, 100 Shih-Chuan 1st Road, Kaohsiung 80708, Taiwan; ^7^Center for Biomarkers and Biotech Drugs, Kaohsiung Medical University, 100 Shih-Chuan 1st Road, Kaohsiung 80708, Taiwan; ^8^School of Pharmacy, China Medical University, 91 Hsueh-Shih Road, Taichung 40402, Taiwan

## Abstract

Glucuronidation is a major metabolism process of detoxification for carcinogens, 4-(methylnitrosamino)-1-(3-pyridy)-1-butanone (NNK) and 1,2-dimethylhydrazine (DMH), of reactive oxygen species (ROS). However, intestinal *E. coli* *β*-glucuronidase (e*β*G) has been considered pivotal to colorectal carcinogenesis. Specific inhibition of e*β*G may prevent reactivating the glucuronide-carcinogen and protect the intestine from ROS-mediated carcinogenesis. In order to develop specific e*β*G inhibitors, we found that 59 candidate compounds obtained from the initial virtual screening had high inhibition specificity against e*β*G but not human *β*G. In particular, we found that compounds **7145** and **4041** with naphthalenylidene-benzenesulfonamide (NYBS) are highly effective and selective to inhibit e*β*G activity. Compound **4041**  (IC_50_ = 2.8 *μ*M) shows a higher inhibiting ability than compound **7145**  (IC_50_ = 31.6 *μ*M) against e*β*G. Furthermore, the molecular docking analysis indicates that compound **4041** has two hydrophobic contacts to residues L361 and I363 in the bacterial loop, but **7145** has one contact to L361. Only compound **4041** can bind to key residue (E413) at active site of e*β*G via hydrogen-bonding interactions. These novel NYBS-based e*β*G specific inhibitors may provide as novel candidate compounds, which specifically inhibit e*β*G to reduce e*β*G-based carcinogenesis and intestinal injury.

## 1. Introduction

Glucuronidation represents a major route of drug metabolism in the liver [1–3]. Many carcinogens and xenobiotics are detoxified by conjugation with a glucuronide acid to increase their water solubility, thus facilitating their excretion [4, 5]. For example, the 4-(methylnitrosamino)-1-(3-pyridy1)-1-butanone (NNK) and 1,2-dimethylhydrazine (DMH), which lead to reactive oxygen species- (ROS-) mediated DNA damage, mutagenesis, and carcinogenesis, can be metabolized and detoxified to glucuronide form by UDP-glucuronosyltransferases (UGTs) [6–8]. However, when the glucuronide conjugates enter the intestine through enterohepatic circulation, they can be hydrolyzed and their toxicity can be reversed by* E. coli β*-glucuronidase (e*β*G) [9–11], which would induce intestinal injury [5, 12–14] and colon carcinogenesis. In addition, chemotherapy-induced diarrhea (CID) and intestinal damage have been clinically demonstrated in several chemotherapeutic drugs such as CPT-11 [15–17], 5-FU [18], and oxaliplatin [19]. There is a specific *β*G inhibitor, glucaro-1,5-lactone, which has been shown to alleviate CPT-11-induced mucosal damage in the small intestine* in vivo *[5]. However, glucaro-1, 5-lactone of the current treatments against CID is limited and not effective, since it preferentially inhibits human *β*G (h*β*G) activity [20], which may induce mucopolysaccharidoses (MPS) [21, 22]. The current treatments against CID are limited and not effective. Therefore, it is crucial to develop an e*β*G specific inhibitor, which cannot affect h*β*G activity, as an effective treatment against carcinogenesis and CID.

The crystal structures of h*β*G and e*β*G have been reported [16, 17]. In addition, e*β*G has a unique “bacteria loop” (LGIGFEAGNKPKELYSE) [16] which is absent in h*β*G. Some known drugs such as Amoxapine [23, 24] and Loxapine [23] and e*β*G inhibitors [16] have been also demonstrated to interact with the residues of bacterial loop and active sites of e*β*G [16, 24]. Those reports indicate that the area around the unique loop and the active site is an important target for e*β*G inhibitor selection.

For the development of specific e*β*G inhibitors, we demonstrated a crystal structure of recombinant e*β*G (provided by Steve R. Roffler, Institute of Biomedical Sciences, Academia Sinica, Taipei, Taiwan) in complex with D-glucaro-1, 5-lactone which revealed that the inhibitor was bound at the residues (E413, E504) of active site. And, we further compared the active center between e*β*G and h*β*G though overleap. We obtained candidate compounds that selectively inhibit e*β*G via computational screening by DOCK 4.0 program [25, 26] and X-ray crystal structure of e*β*G. A chemical database (SPECS) containing ~300,000 commercially available compounds was computationally screened against a grid box enclosing the unique bacterial loop at e*β*G active site. To prove whether the candidate compounds can effectively inhibit e*β*G without affecting h*β*G activity, compounds were examined based on their specific inhibition for e*β*G versus h*β*G by* in vitro β*G activity-based assays. The binding motifs of e*β*G specific inhibitors were determined by molecular docking studies. The novel e*β*G specific inhibitor may provide a highly effective and selective agent to prevent e*β*G-based carcinogenesis and CID.

## 2. Materials and Methods

### 2.1. Expression and Purification of *β*G Protein

Plasmid pRESTB containing *β*G gene and a histidine tag at the N-terminus was constructed as described [27]. Recombinant *β*G (human and* E. coli*) was produced by isopropyl *β*-D-thiogalactopyranoside (IPTG) induction of BL21 (DE3) bacteria. *β*G was purified from bacterial supernatants by affinity chromatography on nickels Sepharose 6 Fast Flow (GE Healthcare). The column was washed by phosphate-buffered saline (PBS), with 50 mM imidazole, and *β*G was eluted by PBS with 250 mM imidazole. The purified *β*G was desalted on a Sephadex G-25 column equilibrated with PBS and stored at −80°C.

### 2.2. Virtual Screening of e*β*G Specific Inhibitors

The virtual screening was performed using the DOCK 4.0 program and the X-ray crystal structure of e*β*G (provide by Steve R. Roffler). The B-chain structure of protein, water molecules, and the cocrystallized inhibitor D-glucaro-1,5-lactone were removed. The remaining A-chain protein structure was used to prepare the target site for docking simulations. The active-site region of e*β*G was specified as the target site for ligand docking in virtual screening. Briefly, a molecular surface around the target site was generated with the MS program using a 1.4 Å probe radius and this surface was used to generate with the SPHGEN program 43 overlapping spheres to fill the target site. A grid box enclosing the target site was created for grid calculations with dimensions 19.3 × 22.4 × 15.6 Å. The force filled scoring grids were calculated with the GRID program using a distance-dependent dielectric constant of 4*r*, an energy cutoff distance of 10 Å, and a grid spacing of 0.3 Å. The database for virtual screening was the SPECS compound collection, which included ~300,000 commercially available compounds (downloaded from the ZINC database web site). The DOCK 4.0 program performs docking simulations using a distance-matching algorithm. The matching parameters used to run virtual screening were set as follows: distance tolerance = 0.5, distance minimum = 2.5, nodes maximum = 10, and nodes minimum = 4. The SPECS database was computationally screened against the active site of e*β*G using the force field scoring function based on interaction energy. Virtual screening was performed on a Silicon Graphics Octane workstation with dual 270 MHz MIPS R12000 processors.

For compound selection, the docking models of the 4724 top-ranked compounds (energy score values ≤−42.00 kcal/mol) were visually inspected using the software PyMOL. Together with consideration of chemical diversity, the selection of compounds was assisted by analysis of the docking models with respect to shape fitting and hydrogen-bonding and hydrophobic interactions. Finally, we selected 59 compounds for enzyme inhibition assays against* E. coli* and human *β*Gs. The compounds for testing were purchased from the SPECS Company. The SPECS ID number and docking energy score for compounds are listed in the supporting information.

### 2.3. *In Vitro* *β*G-Activity Assay of e*β*G Specific Inhibitors

The candidate compounds were purchased from SPECS (The Netherlands). Each candidate was provided as a solid power and dissolved in 100% DMSO (Sigma-Aldrich) to 10 mM as stock. Candidates were screened for their inhibition specificity of e*β*G versus h*β*G, which were conducted at pH 7.3 or pH 5.4, in triplicate, respectively. 40 *μ*L purified *β*G was treated with 10 *μ*L compound solution at 37°C for 30 min and sequentially incubated with 50 *μ*L of pNPG (Sigma-Aldrich) at 37°C for 30 min. Reactions were quenched with 5 *μ*L of 2 N sodium hydroxide (Sigma-Aldrich). Each reaction consisted of 3.75 ng purified *β*G, 50 *μ*M compound, and 5 mM pNPG in PBS containing 10% DMSO and 0.05% BSA (Sigma-Aldrich). *β*G-activities were measured by color development of pNP detected on a microplate reader at OD 405 nm. Results are displayed as percent of *β*G activity compared with the untreated control. For IC_50_ determination, compounds at various concentrations (100 *μ*M to 0.001 *μ*M) were added.

### 2.4. Molecular Docking Studies of e*β*G Specific Inhibitors

The crystal structure of e*β*G for the virtual screening was also utilized in the docking studies of compounds** 7145** and** 4041**. Hydrogen atoms were added to the A-chain protein structure, and the resulting structure was used in the docking simulations. The 3D structures of compounds were built and optimized by energy minimization using the MM2 force field and a minimum RMS gradient of 0.05 in the software Chem3D 6.0 (CambridgeSoft Corp., Cambridge, MA). Docking simulations were performed using the GOLD 5.0 program [28] on an HP xw6600 workstation with Intel Xeon E5450/3.0 GHz Quadcores as the processors. The GOLD program utilizes a genetic algorithm (GA) to perform flexible ligand docking simulations. In the present study, for each of the 30 independent GA runs, a maximum number of 100000 GA operations was performed on a single population of 100 individuals. Operator weights for crossover, mutation, and migration were set to 95, 95, and 10, respectively. The GoldScore fitness function was applied for scoring the docking poses of compounds. The docking region was defined to encompass the active site of e*β*G. The best docking solution (with the highest GOLD fitness score) for a compound was chosen to represent the most favorable predicted binding mode to e*β*G.

## 3. Results

### 3.1. Comparison of the Active Site Structures of e*β*G and h*β*G

**Figure 1:**
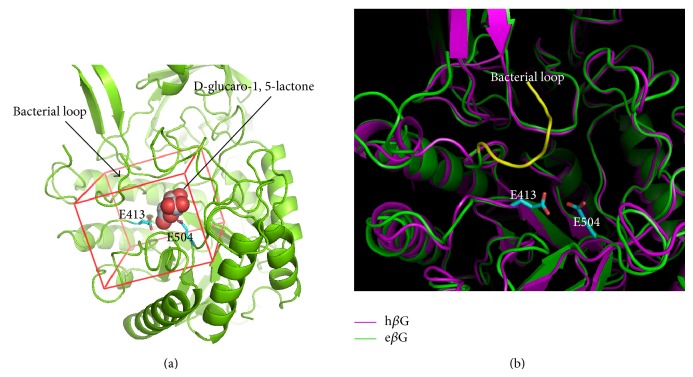
The crystal structure of e*β*G and h*β*G. (a) The crystal structure of e*β*G bound with the inhibitor D-glucaro-1,5-lactone in the active site was used in the virtual screening. A docking box (red line) was defined to enclose the active site for virtual compound screening. (b) The e*β*G (green) and h*β*G (purple) were modeled by superimposing. The e*β*G contains a “bacterial loop” (yellow) not found in the h*β*G. The E413 and E504 are two catalytic residues in the active site of e*β*G.

To identify the active site of e*β*G, recombinant full-length e*β*G was purified and shown to hydrolyze pNPG to PNP for detecting *β*G activity. The enzyme was crystallized in complex with an established inhibitor, D-glucaro-1, 5-lactone. Crystal structure of e*β*G (provided by Steve R. Roffler) in complex with D-glucaro-1, 5-lactone revealed that the inhibitor was bound at residues (E413 and E504) of the active site (Figure 1(a)). To compare the structures of the active centers between e*β*G and h*β*G (PDB ID 1BHG), *β*Gs were analyzed by computer simulation technology. After superimposition, the crystallized structure of e*β*G is 45% similar to h*β*G. Moreover, there is a “bacterial loop” within e*β*G which is absent in h*β*G (Figure 1(b)). Similar results have been also shown in other reports [16]. This e*β*G unique loop of the active center is an ideal target site for screening compounds that can selectively inhibit e*β*G activity.

### 3.2. *In Silico* Virtual Screening of e*β*G Inhibitor Candidates

To identify potential e*β*G inhibitors that can selectively block e*β*G activity, but not h*β*G, the virtual screening proceeded based on the different structures of the active center between e*β*G and h*β*G. The SPECS database (~300,000 commercially available compounds) was computationally screened against the “grid box” which contains the bacterial loop of e*β*G and active site using the DOCK program (version 4.0). Fifty-nine candidate compounds were acquired from the initial virtual screening which was designed to target the bacterial loop of e*β*G and its active site. The docking energy scores of 59 candidate compounds measured by the DOCK program are −43 to −55 kcal/mol (Table S1 in the supplementary material available online at http://dx.doi.org/10.1155/2014/740815).

### 3.3. Screening of e*β*G Inhibitor Candidates by* In Vitro* *β*G Activity Assay

**Figure 2:**
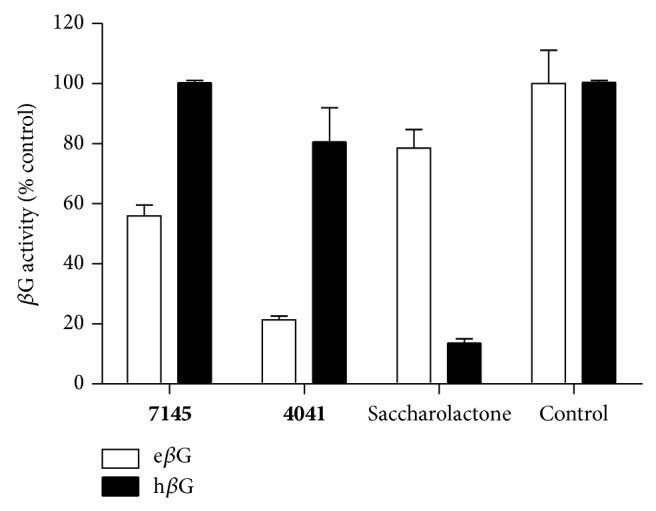
Specific inhibition of compounds** 7145** and** 4041** against e*β*G. Compound** 7145** and compound** 4041** acquired from ligand docking in virtual screening were evaluated based on their selective inhibition for recombinant e*β*G versus h*β*G. 10 *μ*M of compound** 7145**, compound** 4041**, saccharolactone, and 10% DMSO (control) was incubated with purified e*β*G (□) and h*β*G (■), respectively. *β*G activity was determined by hydrolysis of the pNPG substrate. Error bars represent SD; *N* = 3.

To prove whether these 59 candidate compounds can effectively inhibit e*β*G without affecting h*β*G activity, 50 *μ*M compounds were examined for their specific inhibition for e*β*G versus h*β*G by* in vitro β*G-based activity assays, in which the conversion of pNPG to PNP was detected by measuring the increases in PNP absorbance at OD 405 nm. The result showed that all the 59 candidate compounds displayed selective inhibition against e*β*G activity. The inhibiting ability against e*β*G activity, especially, was >95% in 7 candidates of e*β*G specific inhibitors (Table S1). Based on these results, we concluded that the pocket site in the unique loop and active site of e*β*G are an ideal site to screen e*β*G specific inhibitors through virtual screening. We found that compound** 7145** (4-tert-butyl-N-(4-oxo-1(4H)-naphthalenylidene-benzenesulfonamide) can inhibit >95% e*β*G activity and does not hamper h*β*G activity at 50 *μ*M condition (Table S1). The result indicated that the derivatives of naphthalenylidene-benzenesulfonamide (NYBS) might effectively and specifically inhibit the e*β*G activity. Based on the NYBS structure, we performed the substructure search and then found compound** 4041** (4-methyl-N-(4-oxo-3-(1H-1,2,4-triazol-5ylsulfanyl)-1(4H)-naphthalenylidene)benzenesulfonamide). In particular, compound** 4041** has been shown to be a more potent e*β*G antagonist than compound** 7145**.  Figure 2 and Table 1 show that while compound** 4041** (IC_50_ = 2.8 *μ*M) can selectively inhibit >80% e*β*G activity, at 10 *μ*M, the inhibition of compound** 7145** (IC_50_ = 31.6 *μ*M) is 55% inhibition. Compared to D-saccharic acid 1,4-lactone (saccharolactone), which showed higher inhibition on h*β*G, our candidate compounds displayed specificity against e*β*G activity (Figure 2). Based on these results, we concluded that the derivatives of NYBS may provide a novel specific inhibitor to reduce e*β*G-based intestinal injury and CID.

### 3.4. Molecular Docking Studies of e*β*G Specific Inhibitors

**Figure 3:**
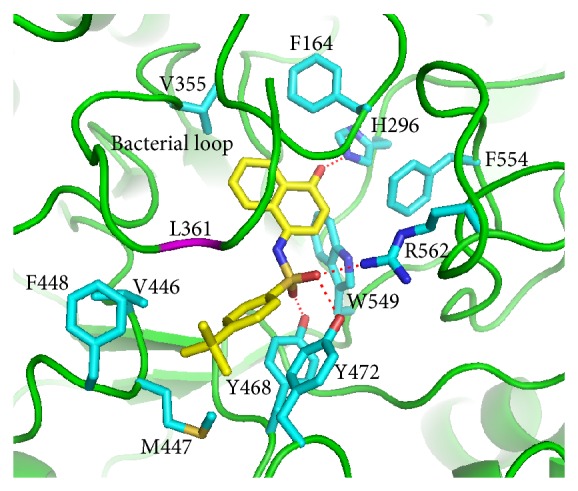
Binding model of compound** 7145**. Predicted binding mode of compound** 7145** in the active site of e*β*G from the docking study. Compound** 7145** (yellow) and some amino acid residues (cyan) interacting with the inhibitor are shown as stick structures. The red dashed lines indicate hydrogen-bonding interactions. The residue L361 (purple) in the bacterial loop makes hydrophobic contact with compound** 7145**.

To predict the binding modes of compounds** 7145** and** 4041** in the active site of e*β*G, we performed molecular docking studies using the GOLD 5.0 program. As depicted in Figure 3, the docking model revealed that compound** 7145** forms four hydrogen bonds to e*β*G. Three of the hydrogen bonds, to residues Y468, Y472, and R562, arise from the SO_2_ group of compound** 7145**. The carbonyl group on the bicyclic 4-oxo-1(4H)-naphthalenylidene ring can form one hydrogen bond with H296. Compound** 7145** makeshydrophobic interactions with the surrounding residues, including W549, F554, F164, V355, V446, M447, F448, Y468, and Y472. The residue L361 in the bacterial loop makes hydrophobic contact with compound** 7145**. Compound** 7145** showed a GOLD fitness score of 62.09.

**Figure 4:**
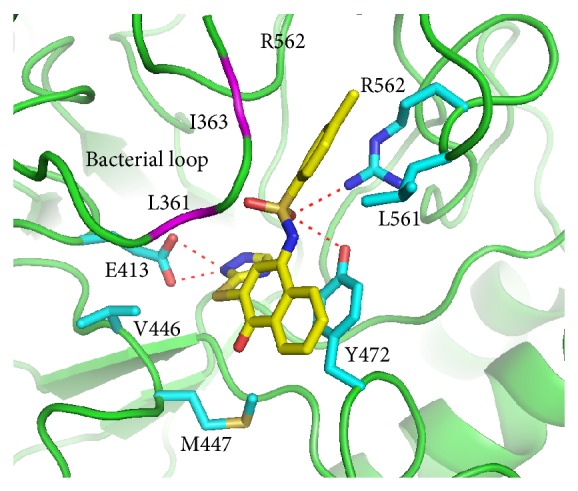
Binding model of compound** 4041**. Predicted binding mode of compound** 4041** in the active site of e*β*G from the docking study. Compound** 4041** (yellow) and some amino acid residues (cyan) interacting with the inhibitor are shown as stick structures. The red dashed lines indicate hydrogen-bonding interactions. The residues L361 and I363 (purple) in the bacterial loop make hydrophobic contact with compound** 4041**.

**Figure 5:**
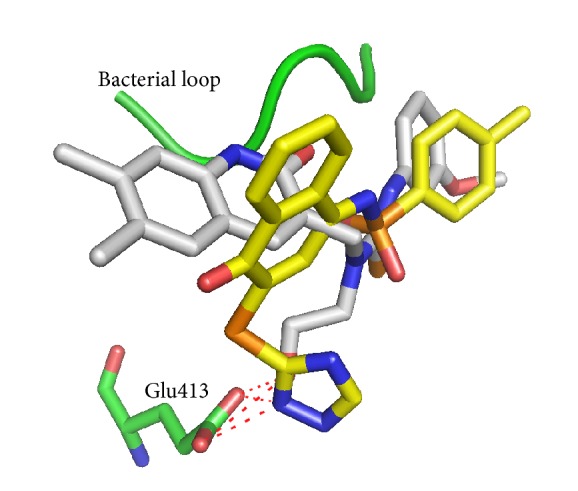
Binding model of compound** 4041** in the crystal structure. Overlay of the docking pose of compound** 4041** (yellow) with the bound orientation of an e*β*G-specific inhibitor (gray) observed in the cocrystal structure of e*β*G.

The docked orientation of compound** 4041** is considerably different from that of compound** 7145** (Figure 3). For compound** 4041**, the bicyclic 4-oxo-1(4H)-naphthalenylidene ring points towards M447 and makes a *π*-*π* stacking interaction with Y472. In contrast, the bicyclic ring of compound** 7145** is oriented in the opposite direction to M447 and located very close to the experimental binding position of the inhibitor D-glucaro-1,5-lactone. Compound** 4041** is hydrogen bonded to residues Y472 and R562 through the SO_2_ group and to E413 through the 1,2,4-triazole moiety. Compound** 4041** makes hydrophobic interactions with the surrounding residues, including V446, M447, Y472, and L561. The residues L361 and I363 in the bacterial loop make hydrophobic contact with compound** 4041** (Figure 4). Compound** 4041** has a GOLD fitness score of 64.91 higher than that of compound** 7145**.  Figure 5 shows an overlay of the docking pose of compound** 4041 **with the bound orientation of an e*β*G-specific inhibitor [16] observed in the cocrystal structure of e*β*G. In comparison to the cocrystallized inhibitor, compound** 4041** has similar binding interactions with e*β*G, including hydrogen bonding to the catalytic residue E413 and close contact with the bacterial loop. Based on these results, we suggest that binding to the active site and the bacterial loop of e*β*G may provide selective abilities of inhibitors against e*β*G.

## 4. Discussion

In this study, we have obtained potent and selective e*β*G inhibitors from* in silico* virtually screening and further confirmed their inhibition specificity by* in vitro β*G activity-based assay. All the 59 candidate compounds from the initial screening showed high effective and selective inhibition against e*β*G. We identified the two most promising compounds, compound** 7145** and its derivate compound** 4041**, showing IC_50_ values of 31.6 *μ*M and 2.8 *μ*M, respectively. Importantly, compound** 4041** with naphthalenylidene-benzenesulfonamide displayed inhibition selectivity against e*β*G by binding to the active site at E413 and the unique loop of e*β*G at L361 and I363.

High-throughput screening (HTS) allows researchers to screen millions of compounds for lead identification in drug discovery. However, this method is limited by the size of compound library. Generally, a compound library is quite costly, and the screening process is time-consuming; thus, the limitations have become more apparent. Hence, virtual screening has become an important tool to access novel drugs for lead indentation [29]. The hit rate of virtual screening can reach up to 2–24% which is much higher than HTS with 0.01–0.001% [30]. In our study, we obtained 59 potential e*β*G inhibitors via virtual screening of a library which consisted of ~300,000 compounds. All candidate compounds showed specific inhibition against e*β*G, but not h*β*G, and met the criteria as virtual screening. The structure-based virtual screening can select compounds with no range limitation and narrow down the candidates for further evaluation, which saves both money and time.

h*β*G is a lysosomal enzyme of normal tissues, and quite low levels of h*β*G are found in serum [31, 32]. In contrast, e*β*G is mainly found in the intestine. Both h*β*G and e*β*G catalyze hydrolysis of *β*-D-glucuronic acid residues from the nonreducing end of glycosaminoglycans [33, 34], but the enzyme has a unique acidic optimum pH. While h*β*G displays maximal catalytic activity at pH 4–4.5 [32, 35], e*β*G exhibits optimal activity at neutral pH. Inhibiting h*β*G may cause MPS [21, 22], a lysosomal storage disease that can affect appearance, physical abilities, organ and system functioning, and, in most cases, mental development. It is crucial to screen compounds that can only block e*β*G activity but not affect h*β*G.

A unique loop structure was found in e*β*G which lacked h*β*G after superimposition of two *β*Gs, which provides a target site to screen compounds that can distinguish the two *β*Gs [16]. We found 59 candidate compounds which can selectively inhibit e*β*G activity through molecular docking against the grid box enclosing the bacterial loop and active site of e*β*G. Some known drugs, such as Amoxapine and Loxapine, have been demonstrated to interact with the residues of bacterial loop and active site of e*β*G and inhibit variant bacterial *β*G activity [24]. Wallace and colleagues indicated that the key residues of bacterial loop are L361 and F365 [16], indicating that we can develop the e*β*G specific inhibitor by targeting the unique loop of e*β*G. In this report, compound** 4041** can bind to E413 (key residue in active site of e*β*G) through the 1,2,4-triazole moiety but not show in the compound** 7145**. Furthermore, compound** 4041** has twohydrophobic contacts to residues L361 and I363 in the bacterial loop. But, compound** 7145** shows one hydrophobic contact withresidue L361. In e*β*G activity assay, compound** 4041** (IC_50_ = 2.8 *μ*M) also shows a higher inhibiting ability than compound** 7145** (IC_50_ = 31.6 *μ*M). We concluded that the inhibiting ability of e*β*G has positive correlation with the interacting quantities to the active site and the unique loop of e*β*G.

e*β*G inhibitors can be developed as a chemotherapy adjuvant to reduce CID [16, 24]. CID is a main side effect that occurs in up to 50–80% of patients depending on chemotherapy regimen [36]. There are several studies indicating that inhibiting *β*G activity can reduce CID and intestinal injury [37]. Inhibition of intestinal *β*G by antibiotics could reduce CPT-11-induced diarrhea* in vivo*. But, antibiotics will kill all native gut floras, including probiotics within the digestive tract, which is not recommended for chemotherapeutic patients. Moreover, the e*β*G has also been considered to play a pivotal role in the development of colon carcinogenesis. For example, the DMH and NNK (ROS based carcinogen) have been report that their glucuronide metabolite may be re-toxic by e*β*G and induce intestinal damage and colon tumor* in vivo* [6–8, 12–14]. e*β*G specific inhibitors may act as colon cancer chemoprevention agents by reducing the generation of xenobiotics from glucuronide metabolites. Thus, the specific e*β*G inhibitor can be applied in nutrient supplement for cancer prevention.

## 5. Conclusions

In conclusion, we have identified that two compounds, compound** 7145** and compound** 4041**, can selectively inhibit e*β*G activity without disrupting h*β*G activity by binding to the active site and the unique loop within e*β*G. Because of their high specificity and efficacy against e*β*G, they have great potential to be developed as a chemotherapy adjuvant for antidiarrhea treatment and cancer chemoprevention agent. Moreover, we proved that inhibitors for the desire enzymes can be selected from virtual screening based on the structure docking showing a high hit rate, which may provide a fast and inexpensive approach for new drug discovery.

## Supplementary Material

Fifty-nine candidate compounds were acquired from the initial virtually screening which was designed to target the bacterial loop of eβG and its active site. The docking energy scores of 59 candidate compounds measured by the DOCK program are -43 to -55 kcal/mol. (Table S1) The candidate compounds were purchased from SPECS (Zoetermeer, The Netherlands). Each candidate was rovided as a solid power and dissolved in 100% DMSO (Sigma-Aldrich, MO, USA) to 10 mM as stock. Candidates were screening for their inhibition specificity of eβG verse hβG, which were conducted at pH 7.3 or pH 5.4 in triplicate, respectively. 40 µL purified βG was treated with 10 µL compound solution at 37 °C for 30 min, and sequentially incubated with 50 µL of pNPG (Sigma-Aldrich) at 37 °C for 30 min. Reactions were quenched with 5 µL of 2 N sodium hydroxide (Sigma-Aldrich). Each reaction consisted of 3.75 ng purified βG, 50 µM compound, and 5 mM pNPG in PBS containing 10% DMSO and 0.05% BSA (Sigma-Aldrich). βG-activities were measured by color development of pNP detected on a microplate reader at OD 405 nm. Results are displayed as percent of βG activity compared with the untreated control. The result showed that all the 59 candidate compounds displayed selective inhibition against eβG activity. Especially, the inhibiting ability against eβG activity was >95% in 7 candidates of eβG specific inhibitors (Table S1).

## Figures and Tables

**Table 1:** The structure, IC_50_, and GOLD fitness scores of compound **7145** and compound **4041** docked into the active site of e*β*G.

Compound	Structure	GOLD fitness score^a^	IC_50_ (*μ*M)
**7145**	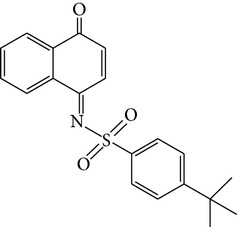	62.09	31.6

**4041**	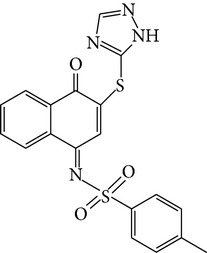	64.91	2.8

^a^Docking simulations were performed using the GOLD 5.0 program.
