# Supplementary material for: Discovery of Specific Inhibitors for Intestinal E. coli β-Glucuronidase through In Silico Virtual Screening
Source: ScientificWorldJournal. 2015 Mar 9;2015:740815. doi: 10.1155/2015/740815 (PMC4370192; doi:10.1155/2015/740815)
Supplement: Supplementary file 1 — Fifty-nine candidate compounds were acquired from the initial virtually screening which was designed to target the bacterial loop of eβG and its active site. The docking energy scores of 59 candidate compounds measured by the DOCK program are -43 to -55 kcal/mol. (Table S1) The candidate compounds were purchased from SPECS (Zoetermeer, The Netherlands). Each candidate was rovided as a solid power and dissolved in 100% DMSO (Sigma-Aldrich, MO, USA) to 10 mM as stock. Candidates were screening for their inhibition specificity of eβG verse hβG, which were conducted at pH 7.3 or pH 5.4 in triplicate, respectively. 40 µL purified βG was treated with 10 µL compound solution at 37 °C for 30 min, and sequentially incubated with 50 µL of pNPG (Sigma-Aldrich) at 37 °C for 30 min. Reactions were quenched with 5 µL of 2 N sodium hydroxide (Sigma-Aldrich). Each reaction consisted of 3.75 ng purified βG, 50 µM compound, and 5 mM pNPG in PBS containing 10% DMSO and 0.05% BSA (Sigma-Aldrich). βG-activities were measured by color development of pNP detected on a microplate reader at OD 405 nm. Results are displayed as percent of βG activity compared with the untreated control. The result showed that all the 59 candidate compounds displayed selective inhibition against eβG activity. Especially, the inhibiting ability against eβG activity was >95% in 7 candidates of eβG specific inhibitors (Table S1). [file 740815.f1.pdf]

Fifty-nine candidate compounds were acquired from the initial virtually screening which was designed to target the bacterial loop of eβG and its active site. The docking energy scores of 59 candidate compounds measured by the DOCK program are -43 to -55 kcal/mol. (Table S1) The candidate compounds were purchased from SPECS (Zoetermeer, The Netherlands). Each candidate was provided as a solid power and dissolved in 100% DMSO (Sigma-Aldrich, MO, USA) to 10 mM as stock. Candidates were screening for their inhibition specificity of eβG verse hβG, which were conducted at pH 7.3 or pH 5.4 in triplicate, respectively. 40 µL purified βG was treated with 10 µL compound solution at 37 °C for 30 min, and sequentially incubated with 50 µL of pNPG (Sigma-Aldrich) at 37 °C for 30 min. Reactions were quenched with 5 µL of 2 N sodium hydroxide (Sigma-Aldrich). Each reaction consisted of 3.75 ng purified βG, 50 µM compound, and 5 mM pNPG in PBS containing 10% DMSO and 0.05% BSA (Sigma-Aldrich). βG-activities were measured by color development of pNP detected on a microplate reader at OD 405 nm. Results are displayed as percent of βG activity compared with the untreated control. The result showed that all the 59 candidate compounds displayed selective inhibition against eβG activity. Especially, the inhibiting ability against eβG activity was >95% in 7 candidates of eβG specific inhibitors (Table S1).

Table S1. The βG activity assay and docking energy score of βG specific inhibitor candidates

	Compound	SPECS ID number	Rank	Docking energy score (kcal/mol)	eβG activity (%) with 50 µM compound	hβG activity (%) with 50 µM compound
1	6368	AK-968/13146368	46	-50.74	44.87	116.03
2	9183	AF-399/42309183	48	-50.67	39.67	107.36
3	5259	AK-968/41025259	167	-48.26	34.05	106.20
4	0136	AQ-405/42300136	289	-47.43	19.16	108.76
5	3043	AG-205/10703043	316	-47.30	25.14	103.55
6	1013	AK-918/12271013	343	-47.16	34.76	126.45
7	3959	AK-968/40733959	428	-46.75	42.88	105.45
8	2715	AG-690/13702715	606	-46.11	53.06	114.38
9	9665	AM-807/42859665	1146	-45.02	8.97	100.83
10	1501	AK-968/41171501	1339	-44.70	27.85	105.12
11	0956	AQ-776/42800956	2155	-43.72	29.70	103.31
12	9107	AG-690/11669107	7	-53.90	29.70	105.37
13	1306	AK-918/13161306	15	-52.64	29.20	105.45
14	5003	AK-968/12345003	99	-49.25	17.02	105.54
15	1046	AF-399/12151046	119	-48.84	45.94	106.69
16	8329	AF-399/40918329	482	-46.59	33.19	97.77
17	8889	AN-329/40718889	483	-46.59	11.40	102.15
18	8010	AG-690/36828010	644	-45.99	23.58	98.84
19	0030	AO-365/15160030	649	-45.98	58.19	111.74
20	8074	AG-690/15438074	773	-45.70	22.15	109.09
21	9572	AQ-750/41789572	833	-45.56	42.66	106.78
22	0523	AN-068/42800523	1142	-45.03	31.77	109.26
23	7145	AQ-390/12597145	1283	-44.79	1.00	119.83
24	6843	AK-968/40946843	1369	-44.66	27.49	108.76
25	8572	AG-389/41558572	1648	-44.26	17.66	102.81
26	4465	AQ-390/40194465	1935	-43.95	10.04	99.50

27	9063	AG-690/33359063	2000	-43.88	32.48	105.45
28	3379	AP-124/43383379	2019	-43.87	31.70	102.56
29	4043	AG-205/36564043	2027	-43.86	32.98	81.24
30	1035	AK-968/06451035	2054	-43.82	25.00	102.48
31	3927	AG-690/12883927	2093	-43.78	18.73	106.86
32	9274	AF-399/15599274	2158	-43.72	32.34	106.69
33	1448	AP-853/42931448	2185	-43.70	11.11	109.67
34	7100	AL-398/12677100	2220	-43.66	20.58	112.56
35	6019	AG-690/36516019	2239	-43.64	33.83	104.96
36	5012	AE-641/00585012	127	-48.76	14.96	108.51
37	0015	AI-204/31730015	180	-48.14	17.59	108.51
38	1135	AO-081/15571135	201	-47.95	3.70	118.10
39	1089	AJ-292/14921089	249	-47.58	47.22	119.17
40	0220	AO-476/43380220	518	-46.43	47.15	113.55
41	4011	AE-641/14824011	626	-46.05	37.54	96.28
42	7670	AP-263/40917670	677	-45.91	43.16	93.31
43	2138	AO-476/40672138	947	-45.37	19.23	107.27
44	7007	AN-919/15527007	1032	-45.21	44.30	105.79
45	5244	AJ-292/41945244	1082	-45.12	26.21	44.38
46	4277	AQ-911/42464277	1247	-44.85	44.37	115.21
47	5033	AG-670/36765033	1412	-44.60	9.33	116.12
48	4021	AE-848/30744021	1441	-44.55	28.56	103.06
49	7036	AF-399/37297036	1628	-44.28	6.70	95.45
50	3250	AO-365/42033250	1667	-44.24	33.62	96.94
51	8013a	AF-407/13078013	1718	-44.17	60.40	100.33
52	3181	AR-434/11133181	1741	-44.14	30.27	88.02
53	8013b	AE-848/33858013	1837	-44.04	32.91	93.72
54	2074	AF-407/13462074	1868	-44.01	12.46	101.07
55	5905	AJ-292/41685905	2048	-43.83	24.93	104.96
56	9927	AN-465/43399927	2209	-43.67	25.00	113.80
57	6015	AO-476/14976015	2331	-43.55	10.40	72.07
58	2020	AJ-292/42032020	2355	-43.53	13.68	84.13
59	6003	AG-690/13506003	2365	-43.52	9.76	93.06
